# Secondary Attack Rate, Transmission and Incubation Periods, and Serial Interval of SARS-CoV-2 Omicron Variant, Spain

**DOI:** 10.3201/eid2806.220158

**Published:** 2022-06

**Authors:** Javier Del Águila-Mejía, Reinhard Wallmann, Jorge Calvo-Montes, Jesús Rodríguez-Lozano, Trinidad Valle-Madrazo, Adrian Aginagalde-Llorente

**Affiliations:** University Hospital of Móstoles, Madrid, Spain (J. Del Águila-Mejía);; Public Health Observatory of Cantabria, Santander, Spain (J. Del Águila-Mejía, A. Aginagalde-Llorente);; Directorate General of Public Health, Government of Cantabria, Santander (R. Wallmann);; Marqués de Valdecilla University Hospital, Santander (J. Calvo-Montes, J. Rodríguez-Lozano, T. Valle-Madrazo)

**Keywords:** COVID-19, incubation period, surveillance, Omicron variant, secondary attack rate, coronavirus disease, SARS-CoV-2, severe acute respiratory syndrome coronavirus 2, viruses, respiratory infections, zoonoses, Spain

## Abstract

Contact tracing data of SARS-CoV-2 Omicron variant cases during December 2021 in Cantabria, Spain, showed increased transmission (secondary attack rate 39%) compared with Delta cases (secondary attack rate 26%), uninfluenced by vaccination status. Incubation and serial interval periods were also reduced. Half of Omicron transmissions happened before symptom onset in the index case-patient.

During December 2021, a total of 622 cases of SARS-CoV-2 infection compatible with the Omicron variant (BA.1/B.1.1.529) (*1*) were studied by the Contact Tracing Programe in Cantabria, Spain. A total of 1,420 close contacts (household, social, and occupational) were identified; 455 secondary cases were identified. We report the main epidemiologic characteristics of these cases, such as secondary attack rate (SAR), transmission period, incubation period, and serial interval, and compared these characteristics with those for Delta variant cases.

## The Study

The Omicron cases were detected among the samples with no amplification of the spike (S) gene (non-S gene target failure) by real-time reverse transcription PCR using the TaqMan SARS-CoV-2 mutation panel (Thermo Fisher Scientific, https://www.thermofisher.com) for single-nucleotide polymorphism genotyping focused on the K417N and L452R mutations. Samples positive for the K417N mutation and negative for L452R were considered compatible with Omicron. The analysis method was validated through whole-genome sequencing of 63 samples. Libraries were constructed by using Ion AmpliSeq SARS-CoV-2 Insight Research Assay and were sequenced with Ion GeneStudio S5 system (both Thermo Fisher Scientific). Next-generation sequencing data were analyzed using Torrent suite software and were assembled by IRMA (*2*). Lineage assignment was done by Pangolin (*3*) by using consensus fasta.

We obtained data on sociodemographic characteristics (age), vaccination status (nonvaccinated or fully vaccinated), and presence or absence of symptoms, as well as symptom onset date (SOD) or diagnosis date (DD) for asymptomatic cases, from the Contact Tracing Program of Cantabria (Appendix). We obtained the same information for the 1,708 coronavirus disease cases of November 2021, when the Delta variant of SARS-CoV-2 represented 100% (1,299/1,299) of samples. We identified 12,587 close contacts and 2,201 secondary cases.

In Spain, close contacts were tested as early as 3 days and as late as 9 days after the date of last contact, depending on when the patient came into the system (*4*). We defined SAR as the proportion of secondary cases among close contacts (those who had been at a distance of <2 m for >15 min) identified through contact tracing (contact 2 days before to 10 days after index case SOD or diagnosis). We classified each relationship by the setting where it took place (household, social, or occupational). We defined global SAR as the average of secondary cases among all relationships (*5*). All SARs (with 95% CIs) are presented by index case-patient’s vaccination status. We tested difference in SAR between Delta and Omicron and differences between SARs for vaccinated and unvaccinated persons by variant and contact setting by Pearson χ^2^ test(Table 1).

**Table 1:** Secondary attack rates of Omicron and Delta variant of SARS-CoV-2, by setting and vaccine status of the index case-patient, Spain*

Settings	Omicron					Delta				Difference, % (95% CI)
	Index cases	Close contacts	Secondary cases	SAR, % (95% CI)		Index cases	Close contacts	Secondary cases	SAR, % (95% CI)	
Global	333	1,126	443	39.3 (36.5–42.2)		1,403	7,013	1,846	26.3 (25.3–27.4)	13† (9.9–16.1)
Unvaccinated index case-patient	210	655	269	41.1 (37.4–44.9)		535	2,876	895	31.1‡ (29.5–32.8)	10† (5.7–14.2)
Vaccinated index case-patient	111	436	159	36.5 (32.1–41.1)		829	3,904	910	23.3‡ (22–24.7)	13.2† (8.3–18)
Household	287	533	263	49.4 (54–53.6)		1,095	2,350	1,129	48 (46–50)	1.3 (−3.4 to 6)
Unvaccinated index case-patient	187	354	171	49.4 (44.2–54.7)		450	1,118	595	53.2‡ (50.3–56)	−3.8 (−9.8 to 2.2)
Vaccinated index case-patient	91	171	85	49.7 (42.3–57)		622	1,198	519	43‡ (40.5–46)	6.4 (−1.6 to 14.4)
Social	143	524	160	30.5 (26.8–34.6)		836	4,153	672	16.2 (15.1–17.3)	14.4† (10.3–18.5)
Unvaccinated index case-patient	76	283	88	31.1 (26–36.7)		315	1,640	284	17.3 (15.6–19.2)	13.8† (7.9–19.7)
Vaccinated index case-patient	61	224	64	28.6 (23.1–34)		495	2,351	368	15.7 (14.2–17.2)	12.9† (6.6–19.3)
Occupational	29	58	18	31 (20.6–43.8)		148	411	43	10.5 (7.93–13.8)	20.6† (7.3–33.8)
Unvaccinated index case-patient	14	22	8	36.4 (19.7–57)		39	97	16	16.5‡ (10.4–25.1)	20.1 (−0.04 to 44.1)
Vaccinated index case-patient	14	34	10	29.4 (16.8–46.1)		105	298	21	7‡ (4.7–10.5)	22.4† (5.1–40)

*SAR, secondary attack rate. †p<0.001. ‡Differences between vaccinated and unvaccinated persons within same-variant context.

Global SAR was 39% (95% CI 36.5%–42.2%) for Omicron cases and 26% (95% CI 25.3%–27.4%) for Delta, a 13-point absolute increase (9.9–16.1; p<0.0001) (Table 1). A higher SAR was also registered in social settings (30.5% for Omicron vs. 16.2% for Delta) and occupational (31% vs. 10.5%) settings but not between household close contacts (49.4% vs. 48%).

Among Delta variant cases, unvaccinated persons showed an overall increased SAR of 7.8% (95% CI 5.6%–10%; p<0.001), household SAR of 9.9% (95% CI 5.8%–14%; p<0.001), and occupational SAR of 9.5% (95% CI 0.8%–18.1%; p = 0.01) compared with vaccinated persons. In contrast, for the Omicron variant, we found no differences between vaccinated and unvaccinated persons in any of these categories.

We selected only symptomatic index cases to calculate transmission, incubation, and serial interval periods. We defined transmission period as the distribution of days from index case SOD to date of last contact with close contacts who became secondary cases. For incubation period and serial interval, we required that the secondary case-patient also be symptomatic. We defined the incubation period as the number of days between date of last contact and secondary case SOD and serial interval as the number of days between the index case SOD and the secondary case SOD (*6*).

**Figure 1:**
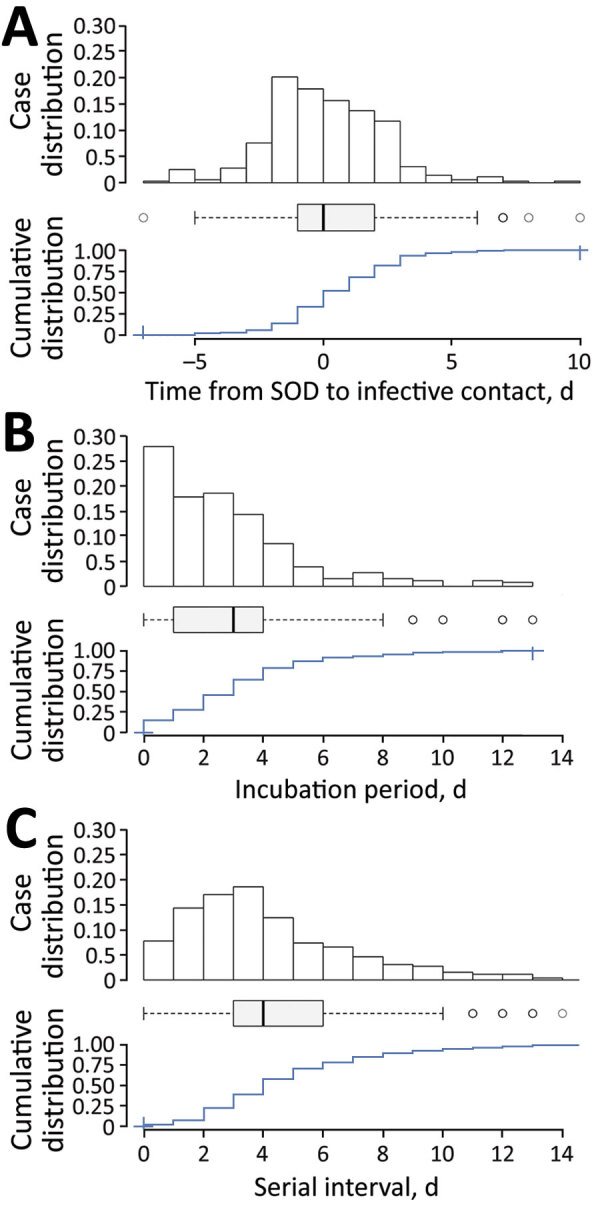
Distribution of Omicron variant SARS-CoV-2 cases, Cantabria, Spain, December 2021. A) Transmission period; B) incubation period; C) serial interval. Each panel shows case density over time (top), a typical boxplot (middle), and cumulative distribution for the period (bottom). For the boxplot, the center line indicates the median, the box left and right ends the interquartile range, the error bars 95% CI, and the open circles outliers. SOD, symptom onset date.

For the 3 periods, we report mean (SD) and median (interquartile range [IQR]). We calculated Omicron-Delta mean differences and Student t test, 95% CI, and p values (Table 2). We constructed histograms, density plots, boxplots, and cumulative distribution functions for Omicron (Figure 1) and Delta (Appendix Figure). 

**Table 2:** Comparison of Omicron and Delta variant of SARS-CoV-2 transmission period, incubation period, and serial interval by index case-patient vaccination status, Spain*

Characteristic	Mean (SD)					Median (IQR)	
	Omicron	Delta	Difference (95% CI)†	p value		Omicron	Delta
Transmission period	0.5 (2.3)	0.8 (2.6)	−0.3 (−0.56 to −0.02)	0.04		0 (−1 to –2)	1 (−1 to 2)
Unvaccinated index case-patient	0.5 (2.3)	0.7 (2.5)	−0.2 (−0.6 to 0.14)	0.22		1 (−1 to 2)	
Vaccinated index case-patient	0.6 (2.3)	0.9 (2.7)	−0.3 (−0.7 to 0.14)	0.89		0 (−1 to 2)	
Incubation period	3.1 (2.6)	3.3 (2.7)	−0.2 (−0.6 to 0.16)	0.29		3 (1–4)	3 (1–5)
Unvaccinated index case-patient	3.1 (2.7)	3.3 (2.6)	−0.2 (−0.7 to 0.3)	0.46		3 (1–4)	
Vaccinated index case-patient	3 (2.2)	3.4 (2.9)	−0.4 (−0.9 to 0.14)	0.16		3 (2–4)	
Serial interval	4.8 (3)	5.4 (3.1)	−0.6 (−1 to −0.15)	0.008		4 (3–6)	5 (3–8)
Unvaccinated index case-patient	4.7 (3.1)	5.4 (3.1)	−0.7 (−1.3 to −0.06)	0.02			5 (3–8)
Vaccinated index case-patient	4.9 (3.1)	5.3 (3.1)	−0.4 (−1 to 0.28)	0.26			5 (3–7)

*IQR, interquartile range. †Student t test for difference in mean of Delta and Omicron period.

**Figure 2:**
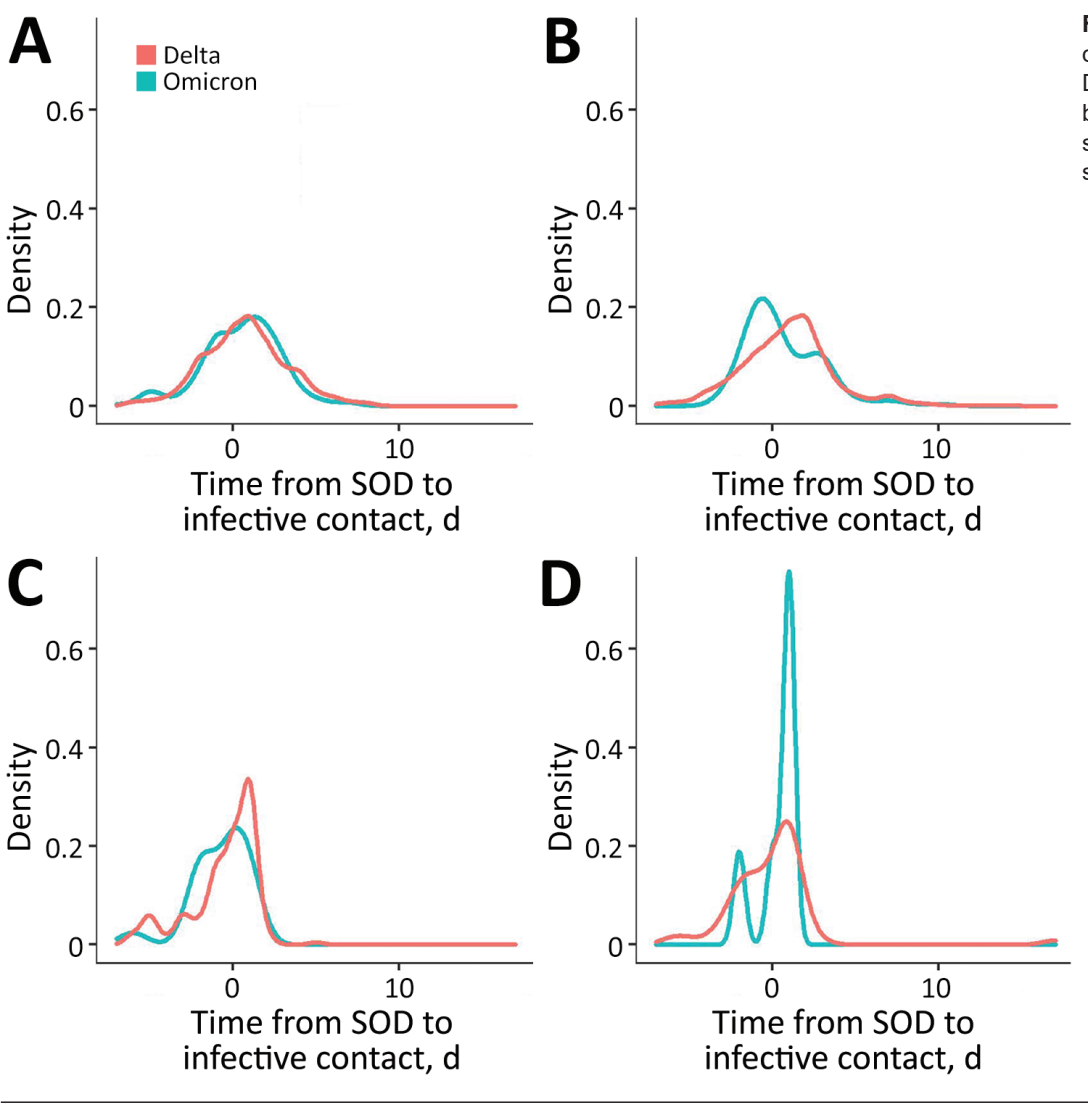
Transmission period distribution for Omicron and Delta variant SARS-CoV-2 cases by vaccination and symptom status, Cantabria, Spain. SOD, symptom onset date.

The transmission period of Omicron cases was shorter (mean 0.5, median 0 days) than Delta cases (mean 0.8, median 1 day) (Figure 1, panel A) and grouped around day 0 after SOD. Mean differences between both variants were significant (−0.3 days; SD −0.56 to −0.02), and IQRs remained equal (Figure 2).

Incubation period had a median of 3 days for both variants and IQR was shorter for Omicron (Figure 1, panel B). We found no mean differences in incubation period. Finally, mean serial interval was significantly shorter for Omicron (4.8 vs. 5.4 days, SD −0.6 to −0.15; p = 0.008) (Figure 1, panel C) with a median of 4 versus 5 days. We found no differences within variants between vaccine status for any of the periods.

## Conclusions

Omicron has spread quickly worldwide since its first notification on November 11, 2021 (*7*). Our findings demonstrate a significant increase in SAR for Omicron cases in Cantabria, Spain, compared with Delta in a similar period and with high vaccine coverage (>80% of target population). Global SAR and social SAR increased by ≈50% (26.3% to 39.3% for global and 16.2% to 30.5% for social), but we did not find significant differences in household SAR. By the end of December, cases increased exponentially, and the Christmas holiday could have affected the number of contacts per case in the occupational and social settings

In this study, vaccinated Omicron index case-patients seemed to have the same transmission capacity as nonvaccinated persons. We did not find this increased transmission capacity for the Delta variant, where significant differences in SAR were observed in global, household, and occupational settings (Table 1) within groups.

Omicron’s increased transmissibility is consistent with the registered tendency of transmission when persons are asymptomatic or early in the symptomatic phase. SARS-CoV-2 transmission took place from day −1 to day +3 of SOD, when most secondary case contacts happen. Median day of transmission was reduced from +1 after symptom onset in Delta to day 0 (SOD) in Omicron (Table 1). Even though the incubation period did not statistically differ, serial interval was significantly decreased in Omicron (mean 4.8 vs. 5.3, median 4 vs. 5) and was again more grouped to the left (IQR 3 vs. 5). Of secondary cases, 90% had an incubation period of 6 days for Omicron and 7 days for Delta.

It has been hypothesized that Omicron’s increased SAR is derived from a concentration of contagion events in the presymptomatic or paucisymptomatic period, when infected persons might be unaware of their status and containment measures such as contact-tracing, isolation, and quick testing are not possible. Half of Omicron contagion events happened before symptom onset. This finding could imply that the effectiveness of nonpharmaceutical measures targeting symptomatic cases (such as contact tracing, quick testing, and isolation) would be substantially decreased in the absence of preventive measures such as social distancing and limiting large gatherings or social meetings.

The social and economic effects of isolation and quarantine have led to continued debate regarding appropriate and adequate quarantine periods, especially in light of possible changes in disease dynamics caused by the Omicron variant (*8*–*13*). In this study, transmission for Omicron and Delta >5 days after SOD was rare, accounting for 8/356 (2%) of secondary cases in Omicron and 79/1,642 (5%) in Delta (Figure 2). This finding could potentially contribute to the debate about quarantine and isolation periods and lessening the social and economic costs of COVID-19 control measures.

This article was preprinted at https://www.researchsquare.com/article/rs-1279005/v1.

Appendix Additional information about secondary attack rate, transmission and incubation periods, and serial interval of SARS-CoV-2 Omicron variant, Spain

## References

[1] World Health Organization. Classification of Omicron (B.1.1.529): SARS-CoV-2 variant of concern [cited 2022 Jan 12]. https://www.who.int/news/item/26-11-2021-classification-of-omicron-(b.1.1.529)-sars-cov-2-variant-of-concern

[2] Shepard SS, Meno S, Bahl J, Wilson MM, Barnes J, Neuhaus E. Viral deep sequencing needs an adaptive approach: IRMA, the iterative refinement meta-assembler. BMC Genomics. 2016;17:708. 10.1186/s12864-016-3030-6 27595578 PMC5011931

[3] O’Toole Á, Scher E, Underwood A, Jackson B, Hill V, McCrone JT, et al. Assignment of epidemiological lineages in an emerging pandemic using the pangolin tool. Virus Evol. 2021;7:veab064. 10.1093/ve/veab064 PMC8344591 34527285

[4] Directorate General of Public Health, Cantabria Health Department. Protocol for detection and management of COVID-19 cases and contacts, version 8 [in Spanish] [cited 2022 Jan 14]. https://www.scsalud.es/documents/2162705/9256837/PROTOCOLO_MANEJO+CASOS+Y+CONTACTOS_V8_221221.pdf/1f7542f9-eca4-5460-1a96-1a121bbca4f5?t=1640176144144

[5] Fung HF, Martinez L, Alarid-Escudero F, Salomon JA, Studdert DM, Andrews JR, et al.; Stanford-CIDE Coronavirus Simulation Model (SC-COSMO) Modeling Group. The household secondary attack rate of severe acute respiratory syndrome coronavirus 2 (SARS-CoV-2): a rapid review. Clin Infect Dis. 2021;73(Suppl 2):S138–45. 10.1093/cid/ciaa1558 33045075 PMC7665336

[6] Alene M, Yismaw L, Assemie MA, Ketema DB, Gietaneh W, Birhan TY. Serial interval and incubation period of COVID-19: a systematic review and meta-analysis. BMC Infect Dis. 2021;21:257. 10.1186/s12879-021-05950-x 33706702 PMC7948654

[7] European Centre for Disease Prevention and Control. Assessment of the further emergence and potential impact of the SARS-CoV-2 Omicron variant of concern in the context of ongoing transmission of the Delta variant of concern in the EU/EEA, 18th update—15 December 2021. Stockholm; The Centre; 2021.

[8] Centers for Disease Control and Prevention. Quarantine and isolation: COVID-19 quarantine and isolation recommendations. 2022 [cited 2022 Jan 19]. https://www.cdc.gov/coronavirus/2019-ncov/your-health/quarantine-isolation.html

[9] European Centre for Disease Prevention and Control. Guidance on quarantine of close contacts to COVID-19 cases and isolation of COVID-19 cases, in the current epidemiological situation, 7 January 2022 [cited 2022 Jan 14]. https://www.ecdc.europa.eu/en/covid-19/prevention-and-control/quarantine-and-isolation

[10] Ministry of Health. Quick risk assessment, SARS-CoV-2 variants in Spain: Omicron, 8th update, December 21, 2021 [in Spanish] [cited 2022 Jan 14]. https://www.sanidad.gob.es/profesionales/saludPublica/ccayes/alertasActual/nCov/documentos/20211221-ERR.pdf

[11] Hay J, Kissler S, Fauver JR, Mack C, Tai CG, Samant RM, et al. Viral dynamics and duration of PCR positivity of the SARS-CoV-2 Omicron variant. 2022 [cited 2022 Jan 19]. https://dash.harvard.edu/handle/1/37370587

[12] National Institute of Infectious Diseases. Active epidemiological investigation on SARS-CoV-2 infection caused by Omicron variant (Pango lineage B.1.1.529) in Japan: preliminary report on infectious period [cited 2022 Jan 19]. https://www.niid.go.jp/niid/en/2019-ncov-e/10884-covid19-66-en.html

[13] Davies M, Bramwell LR, Jeffery N, Bunce B, Lee BP, Knight B, et al. Persistence of clinically relevant levels of SARS-CoV2 envelope gene subgenomic RNAs in non-immunocompromised individuals. Int J Infect Dis. 2022;116:418–25. 10.1016/j.ijid.2021.12.312 34890790 PMC8757659

