# Supplementary material for: Secondary Attack Rate, Transmission and Incubation Periods, and Serial Interval of SARS-CoV-2 Omicron Variant, Spain
Source: Emerg Infect Dis. 2022 Jun;28(6):1224–8. doi: 10.3201/eid2806.220158 (PMC9155885; doi:10.3201/eid2806.220158)
Supplement: Appendix — Additional information about secondary attack rate, transmission and incubation periods, and serial interval of SARS-CoV-2 Omicron variant, Spain [file 22-0158-Techapp-s1.pdf]

Article DOI: https://doi.org/10.3201/eid2806.220158

Secondary Attack Rate, Transmission and Incubation Periods, and Serial Interval of SARS-CoV-2 Omicron Variant, Spain

Appendix

Appendix Table 1. Secondary attack rates of Omicron and Delta variant of SARS-CoV-2 by setting, vaccine status, and number of close contacts per index case, Cantabria, Spain, December 2021

	Omicron				Delta				
Setting	Index cases	Close contacts (C/C)	Secondary cases	SAR (95% CI)	Index cases	Close contacts (C/C)	Secondary cases	SAR (95% CI)	Absolute percentage difference (95% CI)
Global	333	1126 (3.4)	443	39.3% (36.5%–42.2%)	1403	7013 (5)	1846	26.3% (25.3%–27.4%)	13† (9.9–16.1)
Unvaccinated index case-patient	210	655 (3.1)	269	41.1% (37.4%–44.9%)	535	2876 (5.4)	895	31.1%‡ (29.5%–32.8%)	10†* (5.7–14.2)
Vaccinated index case-patient	111	436 (3.9)	159	36.5% (32.1%–41.1%)	829	3904 (4.7)	910	23.3%‡ (22%–24.7%)	13.2† (8.3–18)
Close contacts per index case-patient									
1–2	182	244	103	42% (36%–48%)	532	761	295	39% (35%–42%)	3 (−4–11)
3–4	73	242	109	45% (39%–51%)	375	1279	428	33% (31%–36%)	12† (5–19)
5–6	36	195	71	36% (30%–43%)	192	1048	336	32% (29%–35%)	0.04 (−3–12)
≥7	42	445	160	36% (32%–41%)	304	3925	787	20% (19%–21%)	16† (11–21)
Household	287	533 (1.9)	263	49.4% (54%–53.6%)	1095	2350 (2.2)	1129	48% (46%–50%)	1.3 (−3.4–6)
Unvaccinated index case-patient	187	354 (1.9)	171	49.4% (44.2%–54.7%)	450	1118 (2.5)	595	53.2%‡ (50.3%–56%)	−3.8 (−9.8–2.2)
Vaccinated index case-patient	91	171 (1.9)	85	49.7% (42.3%–57%)	622	1198 (1.9)	519	43%‡ (40.5%–46%)	6.4 (−1.6–14.4)
Close contacts per index case-patient									
1–2	211	285	130	46% (40%–51%)	749	1069	465	43% (41%–46%)	3 (−05–9)
3–4	70	218	118	54% (47%–61%)	283	909	416	46% (43%–49%)	8† (1–16)
5–6	6	30	15	50%	54	295	191	65%	−15

Setting	Omicron				Delta				Absolute percentage difference (95% CI)
	Index cases	Close contacts (C/C)	Secondary cases	SAR (95% CI)	Index cases	Close contacts (C/C)	Secondary cases	SAR (95% CI)	
				(33%–67%)				(59%–70%)	(−35–6)
≥7					9	77	57	74% (63%–83%)	
Social	143	524 (3.7)	160	30.5% (26.8%–34.6%)	836	4153 (5)	672	16.2% (15.1%–17.3%)	14.4† (10.3–18.5)
Unvaccinated index case-patient	76	283 (3.7)	88	31.1% (26%–36.7%)	315	1640 (5.2)	284	17.3% (15.6%–19.2%)	13.8† (7.9–19.7)
Vaccinated index case-patient	61	224 (3.7)	64	28.6% (23.1%–34%)	495	2351 (4.8)	368	15.7% (14.2%–17.2%)	12.9† (6.6–19.3)
Close contacts per index case-patient									
1–2	78	112	28	25% (18%–34%)	374	535	114	21% (18%–25%)	4 (−6 to 13)
3–4	30	102	28	27% (20%–37%)	166	576	117	20% (17%–24%)	7 (−3 to 17)
5–6	17	93	32	34% (26%–45%)	99	536	93	17% (14%–21%)	17† (6–28)
≥7	18	217	72	33% (27%–40%)	197	2506	348	14% (0.13%–0.15%)	19† (13–26)
Occupational	29	58 (2)	18	31% (20.6%–43.8%)	148	411 (2.7)	43	10.5% (7.93%–13.8%)	20.6† (7.3–33.8)
Unvaccinated index case-patient	14	22 (1.6)	8	36.4% (19.7%–57%)	39	97 (2.5)	16	16.5%‡ (10.4%–25.1%)	20.1 (−0.04 to 44.1)
Vaccinated index case-patient	14	34 (2.4)	10	29.4% (16.8%–46.1%)	105	298 (2.8)	21	7%‡ (4.7%–10.5%)	22.4† (5.1–40)
Close contacts per index case-patient									
1–2	21	25	11	44% (27%–63%)	102	131	24	18% (13%–26%)	26† (3–49)
3–4	6	21	2	10% (3%–29%)	24	80	9	11% (6%–20%)	−1 (−18 to 14)
5–6	1	5	2	40% (12%–77%)	11	60	2	3% (1%–11%)	37† (−17 to 91)
≥7	1	7	3	43% (16%–75%)	11	140	8	6% (3%–11%)	37† (−7 to 82)

*Settings are household (living in the same house), occupational (workplace) and social (social relations). Contacts in school, hospitals, and nursing homes were excluded. C/C, contacts per case.
†p<0.000.
‡Differences between vaccinated and unvaccinated persons within same-variant context.

Appendix Table 2. Delta and Omicron cases in Cantabria, Spain, who underwent contact-tracing program by age group and vaccination status

		Delta				Omicron			
Age group, y	Vaccination status	No.	Had symptoms	Hospital Admission	ICU	No.	Had symptoms	Hospital Admission	ICU
<u><</u>11	vaccinated	270	153	3	0	59	41	0	0
12–17	vaccinated	31	19	0	0	31	22	0	0
	unvaccinated	15	9	0	0	5	4	0	0
18–29	vaccinated	45	36	2	1	69	62	0	0
	unvaccinated	62	48	2	1	52	49	0	0
30–39	vaccinated	70	54	2	0	64	56	0	0
	unvaccinated	134	111	2	0	24	21	0	0
40–49	vaccinated	94	72	1	0	89	82	0	0
	unvaccinated	237	189	1	0	36	35	0	1
50–59	vaccinated	63	46	1	0	85	78	0	0
	unvaccinated	217	167	1	1	34	28	0	0
60–69	vaccinated	50	42	6	0	35	31	0	0
	unvaccinated	215	172	10	1	8	6	0	0
70–79	vaccinated	18	16	3	0	6	5	0	0
	unvaccinated	121	91	14	2	2	1	0	0
<u>></u>80	vaccinated	7	3	4	0	12	8	0	0
	unvaccinated	46	27	7	0	2	0	0	0

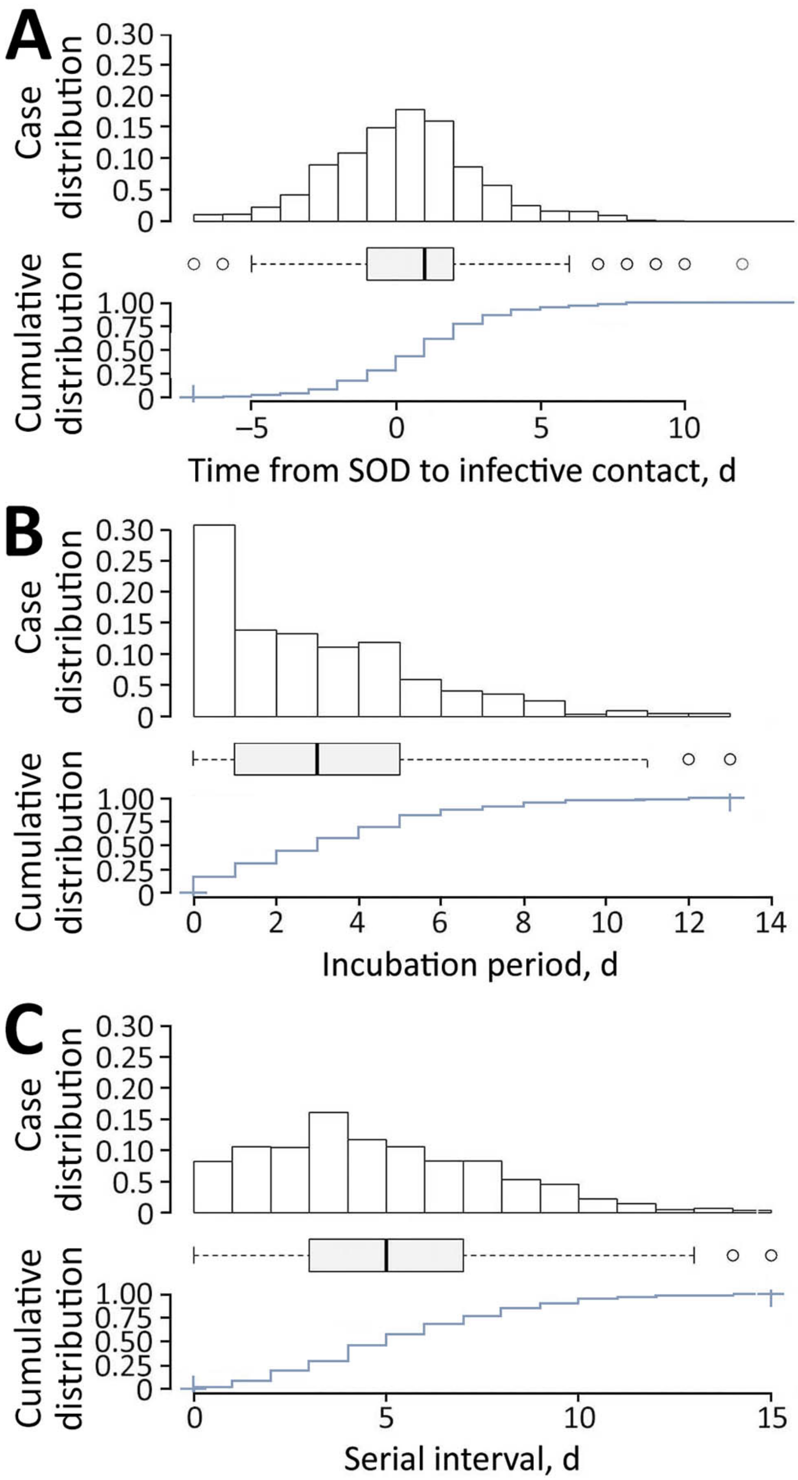

Appendix Figure. A) Transmission period, B) Incubation period, C) Serial Interval of Delta detected cases during November 2021 in Cantabria, Spain. Each period graph includes: density plot for the distribution of the calculated time periods (upper figure), a typical boxplot (middle figure) and a cumulative distribution function graph (bottom figure) with a shared x-axis. A), B) and C) have different x-axes.
